# Preoperative Neutrophil Lymphocyte Ratio Can Be Used as a Predictor of Prognosis in Patients With Adenocarcinoma of the Esophagogastric Junction: A Systematic Review and Meta Analysis

**DOI:** 10.3389/fonc.2020.00178

**Published:** 2020-02-21

**Authors:** Xiao-bo Liu, Zi-ye Gao, Qing-hui Zhang, Sandeep Pandey, Bo Gao, Fan Yang, Qiang Tong, Sheng-bao Li

**Affiliations:** ^1^Department of Gastroenterology, Taihe Hospital, Hubei University of Medicine, Shiyan, China; ^2^Department of Oncology, Taihe Hospital, Hubei University of Medicine, Shiyan, China; ^3^Post Graduate Department, Hubei University of Medicine, Shiyan, China; ^4^Department of Laboratory Medicine, Taihe Hospital, Hubei University of Medicine, Shiyan, China

**Keywords:** AEG, NLR, PLR, prognosis, meta-analysis

## Abstract

**Objective:** Neutrophil lymphocyte ratio (NLR), Lymphocyte mononuclear cell ratio (LMR), and Platelet lymphocyte ratio (PLR) can be used as various prognostic factors for malignant tumors, but the value of prognosis for patients with adenocarcinoma of the esophagogastric junction (AEG) has not been determined. This study used meta-analysis to assess the value of these indicators in the evaluation of AEG prognosis.

**Methods:** Relevant literatures on the prognostic relationship between NLR, LMR, PLR, and AEG was retrieved from PubMed, Web of Science, Embase, Cochrane Library, Cochrane Central Register of Controlled Trials, Wanfang data, and Chinese National Knowledge Infrastructure. The search time from database establishment to June 30, 2019. The language is limited to English and Chinese. Data was analyzed using Stata 15.0 software.

**Result:** Six retrospective studies were included, five of them involved NLR and six of them involved PLR. No LMR literature that adequately satisfied the conditions was retrieved. Increased NLR was significantly associated with a significant reduction in overall survival (OS), cancer-specific survival (CSS), or disease specific survival (DSS) in patients with AEG [hazard ratio (HR) = 1.545, 95% CI: 1.096–2.179, *P* < 0.05]. Subgroup analysis showed that NLR had significant value in the prognosis of both Chinese and Non-Chinese patients (*P* = 0.009 vs. *P* = 0.000). NLR had significant prognostic value for ≥3 and <3 groups (*P* = 0.022 vs. *P* = 0.000). NLR has a significant prognostic value for samples ≥500 and <500 (*P* = 0.000 vs. *P* = 0.022). NLR and OS/CSS/DSS single factor meta-regression showed that regional NLR cut-off values and sample size may be the source of heterogeneity in AEG patients (all *P* < 0.05). There was no significant association between elevated PLR and OS in patients with AEG (HR = 1.117, 95% CI: 0.960–1.300, *P* > 0.05). PLR had no significant prognostic value for both Chinese and UK patients (*P* = 0.282 vs. *P* = 0.429). PLR had no significant prognostic value for ≥150 group and <150 group (*P* = 0.141 and *P* = 0.724). No significant prognostic value was found in either the 300 group and <300 group (*P* = 0.282 vs. *P* = 0.429).

**Conclusion:** Preoperative NLR rise was an adverse prognostic indicator of AEG. High-risk patients should be treated promptly. The results showed that PLR was not recommended as a prognostic indicator of AEG.

## Introduction

Esophagogastric junction (EGJ) cancer mainly refers to cancer whose center of the malignant tumors is within 5 cm of the proximal and distal ends of EGJ, including EGJ distal esophageal adenocarcinoma, cardiac cancer, and proximal gastric cancer (1). In recent years, trend of EGJ cancer is increasing yearly in Europe, the United States, and many Asian countries (2–6) and has become a worldwide problem that seriously endangers human health (7). The adenocarcinoma of the esophagogastric junction (AEG), first proposed by Siewert (8) in 1999, is a tumor with unique clinicopathological features and biological behavior. AEG lymph node metastasis has a high incidence and a low long-term survival rate, affecting the prognosis of patients seriously (9, 10).

Effective markers are screened to identify high-risk patients and helpful for the individualized treatment and prognosis improvement of AEG. Xu et al. (11) reported that log odds of positive lymph nodes can predict the prognosis of patients with Siewert type II AEG. Felismino et al. (12) believes that the prognosis of locally advanced esophagogastric cancer can be determined by pathological staging and primary site. Kudou et al. (13) believes that postoperative sarcopenia can be used as a prognostic indicator for AEG, but these indicators are postoperative. Unfortunately, effective preoperative biomarkers are still lacking.

Laboratory indicators may be used as prognostic indicators for gastrointestinal tumors (14, 15), including: neutrophil to lymphocyte ratio (NLR), platelet to lymphocyte ratio (PLR), lymphocyte monocyte ratio (LMR), Glasgow prognostic score (GPS), and Prognostic nutritional index (PNI). Although some studies have reported the relationship between these indicators and the prognosis of patients with AEG, a consensus has not been reached. Urabe et al. (14) indicated that preoperative NLR and PLR is associated with OS and DFS in patients with AEG. Zhou et al. (16) postulated that preoperative LMR and PLR are very useful predictors for AEG surgery; but Zhang et al. (17) reported that preoperative NLR can be used as prognostic factor for Siewert type II/III AEG patients, but PLR value is limited. The present study aims to evaluate the value of NLR, LMR, and PLR in evaluating the prognosis of patients with AEG through systematic review and meta-analysis and to provide evidence-based supporting the use of these markers as prognostic indicators of AEG.

## Materials and Methods

### Literature Search Strategy

Search for the relationship between NLR, PLR, LMR, and AEG prognosis or clinicopathological features in databases such as PubMed, Web of Science, Embase, Cochrane Library, Cochrane Central Register of Controlled Trials, Wanfang data, and Chinese National Knowledge Infrastructure. The search time frame for database establishment was till June 30, 2019. Search terms included: “NLR,” “PLR,” “LMR,” “esophagogastric junction cancer,” and “AEG.” The language was limited to English and Chinese.

### Inclusion and Exclusion Criteria

Inclusion criteria: (1) Patients confirmed pathologically as AEG; (2) Assessed preoperative NLR, PLR, or LMR overall survival (OS), disease-free survival (DFS), tumor-specific survival (relationship between cancer specific survival (CSS), or disease specific survival (DSS); (3) Reported hazard ratio (HR) and 95% confidence interval (CI) or indirect calculation HR and 95% CI. If HR could not be directly extracted from the literature, we calculated it by formula. Our calculation formula is b = In (HR), stderr = b/inverse-normal-distribution (P/2), 95% CI = exp (b ± 1.96 ^*^ stderr). (4) Full text in English or Chinese.

Exclusion criteria: (1) HR and 95% CI cannot be obtained directly or indirectly, NLR, PLR, or LMR have no clear cut-off point; (2) Non-research literature such as review, case report, conference summary, etc.; (3) Animal research or basic cell research; (4) Repeated published literature, research literature on the same cohort study subjects.

### Test Screening and Data Extraction

We conducted this study in accordance with the systematic reporting and preliminary analysis items (18) (preferred reporting items for systematic reviews and meta analyses, PRISMA). Since all data are based on published literature, this study does not address any ethical issues. Two reviewers (Liu and Zhang) read the title and abstract independently in a double-blind manner, excluded non-compliant studies, read full-text documents that met the inclusion criteria, and cross-checked the included articles. In case of differences and discussions, an independent third party was asked to decide. Data extraction content was based on: title, author, region, publication time, sample size, critical value, HR, and 95% CI of OS, DFS, CSS, or DSS.

### Quality Evaluation

The quality of the included studies was assessed using the Newcastle-Ottawa Quality Assessment Scale (NOS). NOS consists of three aspects: selection, comparability, and exposure or outcome; a total score of 9 points, and a total score of ≥6 in the study is considered high quality (19).

### Statistical Methods

Meta-analysis was performed using Stata statistical software (Stata Corporation, version 15.0, College Station, TX, USA). HR and 95% CI were combined in the study to evaluate the value of NLR, PLR, and LMR in predicting the prognosis of patients with AEG. A meta-analysis forest map that plots the effect indicators. The *Q*-test and I^2^ of the chi-square test were used to evaluate the heterogeneity between studies. I^2^ and *Q*-tests were used to evaluate the heterogeneity of the included research questions. If I^2^ <50% and *P-*test of *P* > 0.1, it indicates that the studies are homogeneous and a fixed effect model is selected; *P* < 0.1, indicating that there is heterogeneity between studies, and a random effect model was selected (19, 20). To find the sources of heterogeneity, subgroup analyses were used to explore sources of heterogeneity. The publication bias was assessed by Egger test and Begg test. When *P* < 0.05, the difference was considered statistically significant and there was publication bias.

## Results

### Characteristics of the Included Literature

**Figure 1:**
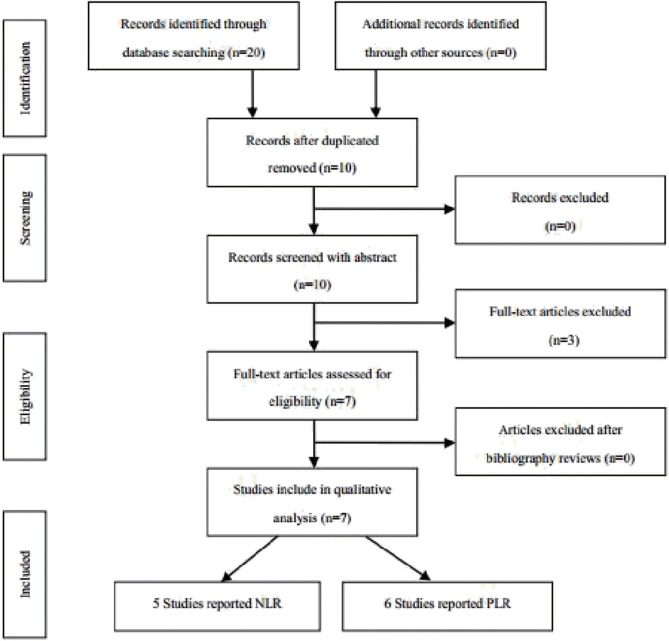
Flow chart of study selection for inclusion in the meta-analysis.

According to the above strategy, a total of 20 articles were screened and finally included in six articles (Figure 1), all of which were retrospective studies. Five of which involved NLR and six of which involved PLR. The information on the literature included in this study (Table 1).

**Table 1:** Basic information of included studies.

**References**	**Ethnicity** **(country)**	**No** **(male/female)**	**Age**	**Stage**	**NLR cutoff value**	**PLR cutoff value**	**LMR cutoff value**	**Outcome**	**Fellow-up** **(months)**	**NOS**
Zhang et al. (21)	Asia (China)	355 (281/74)	64	I–IV	3.5	171	NR	OS	52	7
Zhang et al. (17)	Asia (China)	641 (488/123)	63	I–III	2.22	124.4	NR	CSS	72	7
Zhou et al. (16)	Asia (China)	309 (249/60)	63	I–III	1.697	96.960	NR	OS	51.4	6
Wang et al. (22)	North America (USA)	1,498 (929/569)	66	T_0−4_N_0−3_M_0_	2.8	NR	NR	DSS	48	8
Messager et al. (23)	Europe (UK)	153 (128/25)	64.9	T_0−4_N_0−3_M_0_	NR	192	NR	OS, DFS	31.8	8
Yuan et al. (24)	Asia (China)	327 (282/45)	63.1	I–IV	5	150;300	NR	OS, DFS	24.7	7
Dutta et al. (25)	Europe (UK)	112 (85/27)	NR	I–IV	2.5; 5	150; 300	NR	CSS	55	7

*NLR, neutrophil to lymphocyte ratio; PLR, platelet to lymphocyte ratio; OS, overall survival; CSS, cancer-specific survival; DSS, disease-specific survival; DFS, disease-free survival; NR, not reported*.

### Meta Analysis Results

#### The Prognostic Role of NLR in AEG

**Figure 2:**
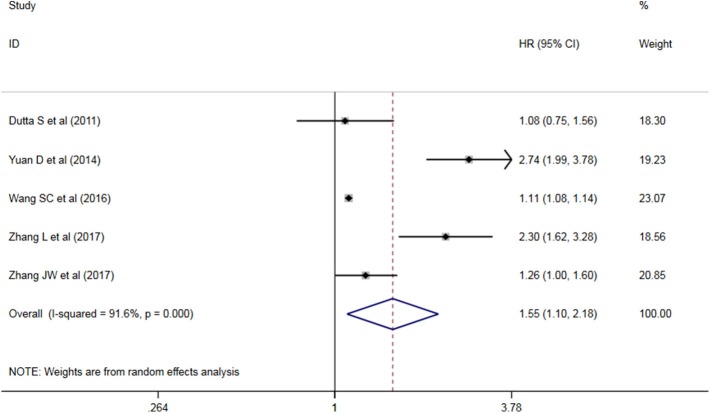
Forest map of the relationship between NLR and OS/CSS/DSS in patients with esophageo-gastric junction cancer.

Five studies have analyzed the relationship between NLR and OS/CSS/DSS in patients with AEG, and a significant heterogeneity exists among these studies (*P* < 0.05, I^2^ = 91.6%), and random effects model was used. These results indicate that an increase in NLR decreases predicts OS/CSS/DSS shortening in patients with AEG (HR = 1.545, 95% CI: 1.96–2.179, *P* < 0.05, Figure 2).

#### The Prognostic Role of PLR in AEG

**Figure 3:**
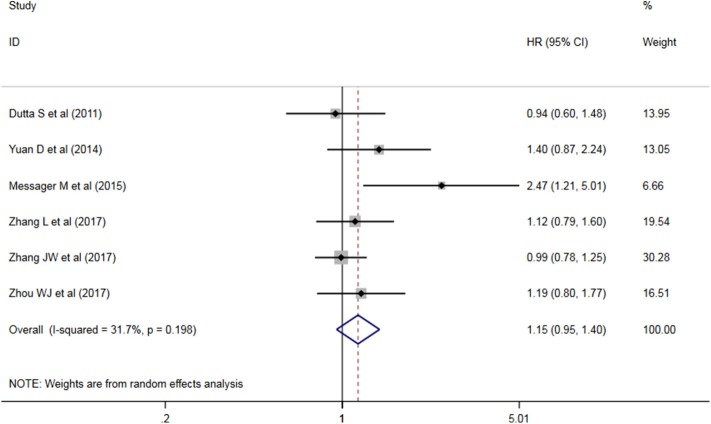
Forest map of PLR and AEG patient OS.

**Figure 4:**
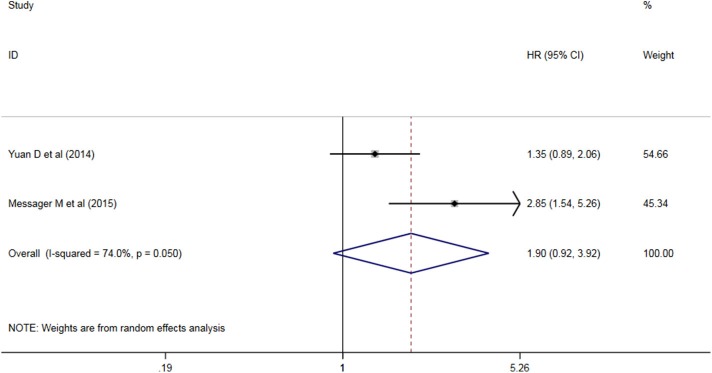
Forest map of DLR relationship between PLR and AEG patients.

Six studies analyzed the relationship between PLR levels and OS in patients with AEG. There was no significant heterogeneity between the studies (*P* = 0.198, I^2^ = 31.7%), so a fixed effect model was used. The results showed that PLR was not suitable as an OS judgment index for AEG patients (HR = 1.117, 95% CI: 0.960–1.300, *P* > 0.05, Figure 3). Two studies analyzed the relationship between PLR levels and DFS in patients with AEG. There was significant heterogeneity between the studies (*P* = 0.050, I^2^ = 74.0%), and random effects model was used for analysis. The results showed that PLR was not suitable as a predictor of DFS in patients with AEG (HR = 1.90, 95% CI: 0.92–3.92, *P* > 0.05, Figure 4).

### Subgroup Analysis Results

#### Heterogeneity Between NLR Studies

**Figure 5:**
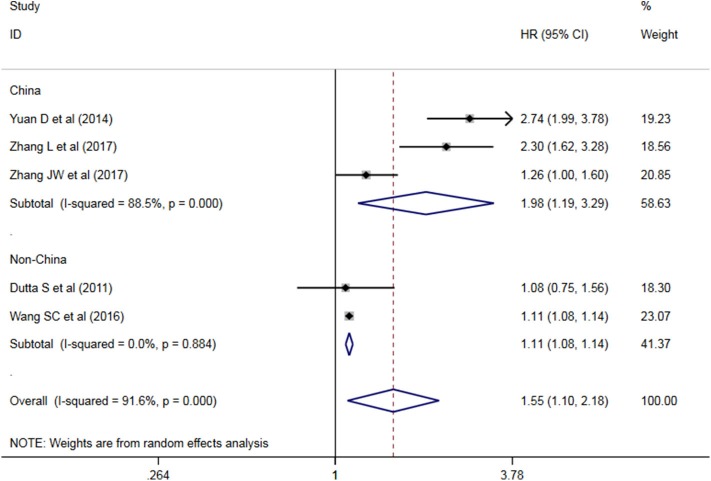
Meta-analysis of NLR and OS/CSS/DSS in Chinese and non-Chinese subgroup AEG patients.

**Figure 6:**
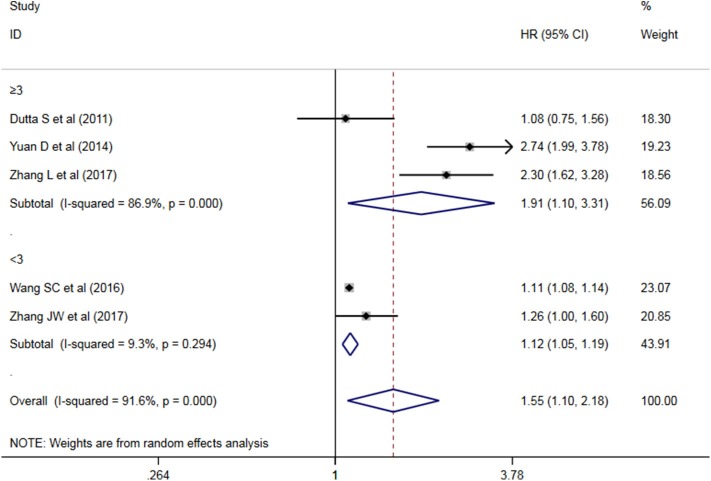
Meta-analysis forest map of NLR ≥3 vs. NLR <3 subgroup overall OS/CSS/DSS.

**Figure 7:**
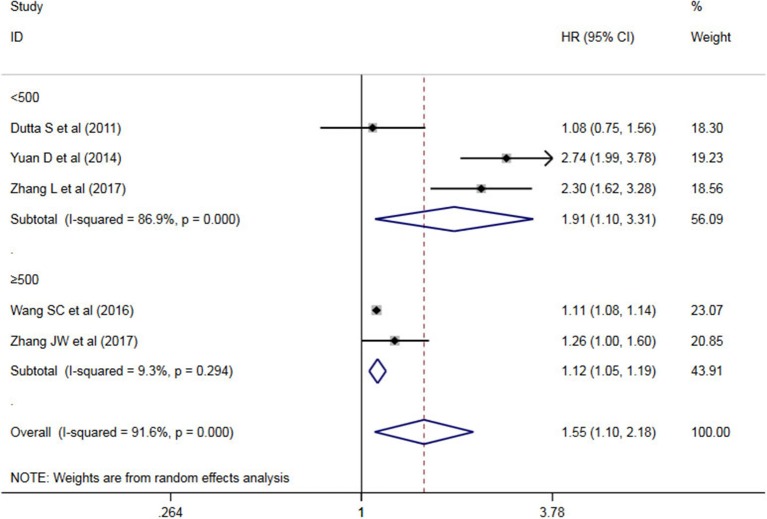
Sample ≥500 group vs. <500 group AEG patient NLR and OS/CSS/DSS meta-analysis forest map.

A subgroup analysis of heterogeneity sources between OS/CSS/DSS studies of NLR and AEG patients. NLR had significant value in the prognosis of patients in China and Non-China (*P* = 0.009 vs. *P* = 0.000, Figure 5); China group had significant heterogeneity (*P* = 0.000), but Non-China group did not have significant heterogeneity (*P* = 0.884). NLR had significant prognostic value for cutoff value ≥3 group and <3 group (*P* = 0.022 vs. *P* = 0.000, Figure 6); ≥3 group had significant heterogeneity (*P* = 0.000), <3 group was not significant heterogeneity (*P* = 0.294). NLR had significant prognostic value for samples ≥500 group and <500 group (*P* = 0.000 vs. *P* = 0.022, Figure 7), ≥500 group had no significant heterogeneity (*P* = 0.294), while <500 group had significant heterogeneity Sex (*P* = 0.000). The results are presented in Table 2.

**Table 2:** Meta-analysis results of NLR and OS/CSS/DSS in patients with carcinoma of the esophagogastric junction.

**Factor**	**No. of study**	**No. of patients**	**HR (95% CI), *P***	**I^**2**^ (%), *P***
NLR				
Overall	5	2,933	1.545 (1.096–2.179), 0.013	91.6%, 0.000
**SUBGROUP ANALYSIS**				
**Country**				
China	3	1,323	1.975 (1.185–3.293), 0.009	88.5%, 0.000
Non-China	2	1,610	1.545 (1.096–2.179), 0.000	0.0%, 0.884
**Cutoff value**				
≥3	3	749	1.906 (1.098–3.309), 0.022	86.9%, 0.000
<3	2	2,139	1.118 (1.055–1.185), 0.000	9.3%, 0.294
**Sample size**				
≥500	2	2,139	1.118 (1.055–1.185), 0.000	9.3%, 0.294
<500	3	749	1.906 (1.098–3.309), 0.022	86.9%, 0.000

#### Heterogeneity Between PLR Studies

**Figure 8:**
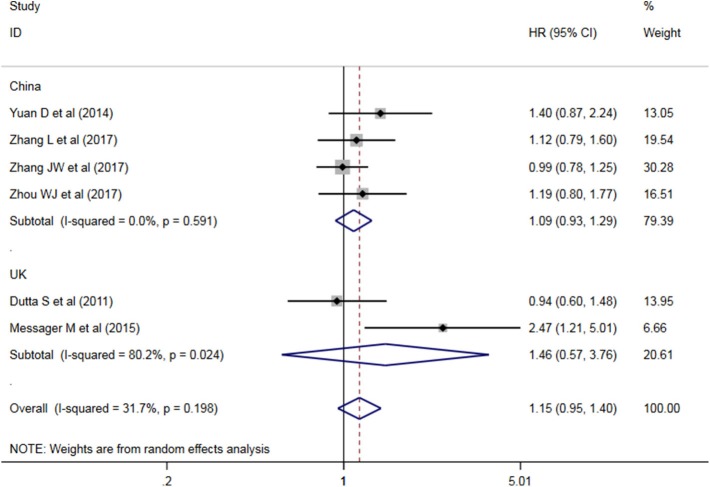
Meta-analysis of PLR and DFS in Chinese and non-Chinese subgroup AEG patients.

**Figure 9:**
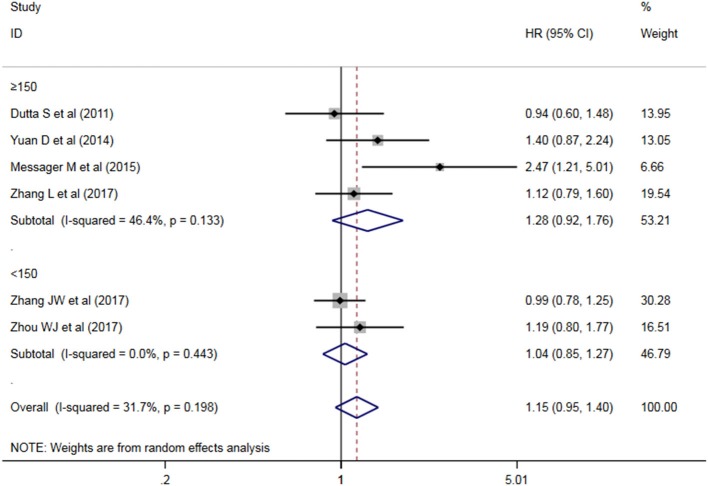
Meta-analysis of the PLR ≥150 vs. PLR <150 sub-group DFS.

**Figure 10:**
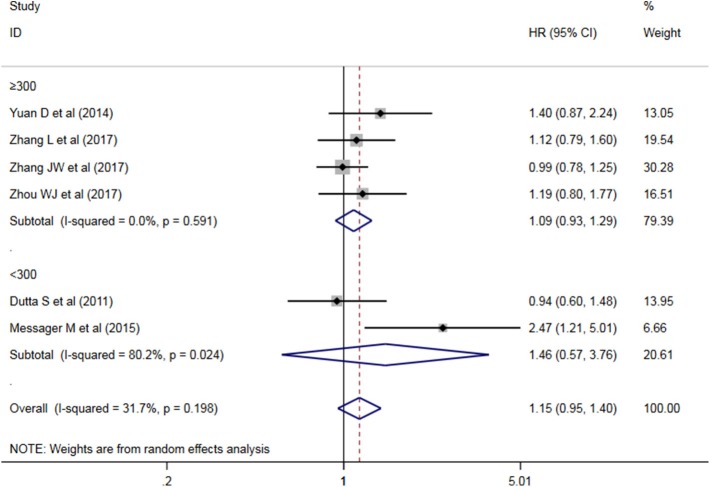
Sample ≥300 group vs. <300 group AEG patient PLR and DFS meta-analysis forest map.

A subgroup analysis of heterogeneity sources between OS studies of PLR and AEG patients. There was no significant heterogeneity in the China group (*P* = 0.591) (Figure 8), and there was significant heterogeneity in the UK group (*P* = 0.024). PLR had no significant prognostic value for Cutoff value ≥150 group and <150 group (*P* = 0.141 vs. *P* = 0.724, Figure 9); there was no significant heterogeneity between the two groups (*P* = 0.133 and *P* = 0.443). PLR had no significant prognostic value for ≥300 group and <300 group (*P* =0.282 vs. *P* =0.429, Figure 10); ≥300 group had no significant heterogeneity (*P* = 0.591), <300 group was significantly heterogeneous Sex (*P* = 0.024). The results are presented in Table 3.

**Table 3:** Meta-analysis results of PLR and DFS in patients with carcinoma of the esophagogastric junction.

**Factor**	**No. of study**	**No. of patients**	**HR (95% CI), *P***	**I^**2**^ (%), *P***
Overall	6	1,897	1.117 (0.960–1.300), 0.153	31.7%, 0.198
**SUBGROUP ANALYSIS**				
**Country**				
China	4	1,632	1.095 (0.928–1.292), 0.282	0.0%, 0.591
UK	2	265	1.463 (0.570–3.759), 0.429	80.2%, 0.024
**Cutoff value**				
≥150	4	947	1.275 (0.923–1.762), 0.141	46.4%, 0.133
<150	2	950	1.037 (0.846–1.271), 0.724	0.0%, 0.443
**Sample size**				
≥300	4	1,632	1.095 (0.928–1.292), 0.282	0.0%, 0.591
<300	2	265	1.463 (0.570–3.759), 0.429	80.2%, 0.024

NLR shows a significant prognostic value in Chinese and non-Chinese patients. A significant heterogeneity was also observed in the Chinese group but not in the non-Chinese group. Meta-regression analysis was conducted to determine the factors that might cause heterogeneity. The variables included region and sample size. NLR and OS/CSS/DSS meta-regression analysis showed that the region might be the source of AEG heterogeneity.

### Meta-Review Analysis

To find factors that may cause heterogeneity, we used meta-regression analysis, the variables included region, cut-off value, and sample size. The NLR and OS/CSS/DSS single factor meta-regression showed that the region, cut-off value, and sample size were all possible reason. It is the source of heterogeneity in patients with AEG (all *P* < 0.05, Table 4).

**Table 4:** Univariate meta regression analysis of NLR and OS/CSS/DSS.

**Variable**	**Coefficient**	**Standard error**	***P*-value**
Region	−0.4728393	0.0863413	0.000
Cutoff value	−0.5717037	0.1025993	0.000
Sample size	−0.5717037	0.1025993	0.000

### Risk of Bias

Begger's test and Egger's test were used to evaluate the published bias, and the results showed that the NLR published bias test (*P*_Begg_ = 0.806 vs. *P*_Egger_ = 0.141) revealed no significant bias.

## Discussion

Infection may involve the entire process of tissue carcinogenesis, directly or indirectly affecting its development (26). Systemic inflammatory response is associated with the inhibition of apoptosis, angiogenesis and DNA damage, leading to tumor progression and metastasis (27). Although the mechanism between hematological parameters and tumors remains unclear, their correlation can be explained by infiltrating immune cells and inflammatory proteins (28). A tumor microenvironment contains many different mediators. Neutrophils promote tumor development (29), cytokine production, and provide a microenvironment for tumor survival. Neutrophils can promote the production of a variety of inflammatory cytokines, providing a good microenvironment for tumor survival and proliferation. On the contrary, lymphocytes play an important role in tumor-specific immune response.

As an independent factor, the effect of chronic inflammation on gastrointestinal cancer has been demonstrated (30). The level of NLR may reflect the inflammatory state of the body. Neutrophils can promote tumor growth and progression by increasing the concentration of some inflammatory substances, such as vascular endothelial growth factor, interleukin-6, and IL-1 (31, 32).

In addition, increased neutrophils inhibit the lysis activity of some cells, such as lymphocytes, natural killer cells and activated T lymphocytes. Lymphocytes play an immunity-related role in tumors. Cytokines released and their mediated cytotoxic by lymphocytes can inhibit cell proliferation and metastasis. However, cytokines produced by cells can lead to lymphocyte depletion and decrease the anti-tumor effect of lymphocytes. As a result, the risks of recurrence and metastasis are increased, leading to poor prognosis (33).

Relative lymphocyte reduction may reduce lymphocyte-mediated anti-tumor cellular immune responses. Platelet aggregation promotes adhesion and aggregation of circulating tumor cells, which enhances the ability of tumor cells to escape immune attack (34). In addition, activated platelets release more vascular endothelial growth factor and a variety of cytokines, thereby increasing the angiogenesis of tumor tissue and ultimately promoting its growth (35, 36). Therefore, in the current situation of the lack of more reliable tumor prognostic indicators, NLR and PLR may provide information on patient prognosis. It is currently known that NLR and/or PLR may be associated with a variety of tumor prognosis, including non-digestive tumor NSCLC (37), breast cancer (38), ovarian cancer (39), Hodgkin lymphoma (40), prostate cancer (41), cervical cancer (42), nasopharyngeal carcinoma (28), and tumors of the digestive tract such as esophageal squamous cell carcinoma (43, 44), gastric cancer (45), pancreatic cancer (46), and colorectal cancer (47).

As far as we know, our research involves the first meta-analysis of the value of the above indicators in the diagnosis of AEG. In this study, 2,933 and 1,897 patients with AEG were included to investigate the prognostic value of NLR and PLR for AEG. The meta-analysis showed that NLR can be used as a prognostic indicator for patients with AEG, but PLR may not be suitable for the OS and DFS of patients with AEG as indicators. Similarly, NLR has significant prognostic value in each subgroup, but PLR has no significant prognostic difference in each subgroup. NLR and OS/CSS/DSS univariate meta-regression showed that regions, NLR cutoffs, and sample sizes may be sources of heterogeneity in patients with AEG, while PLR and OS/CSS univariate metasindicate that the regions, cutoffs, and sample sizes are not possible sources of heterogeneity in patients with AEG. Despite great efforts to obtain relevant research, some data that are not published online are still not available. Hence, more studies should be included in the later stage to reduce heterogeneity.

This study has the following limitations. First, despite we were very cautious about the literature included to draw our conclusions, the number of included studies is not large and covers only Chinese and English literature. Second, the selected literatures are retrospective studies, lacking a prospective cohort study, may result in analytical bias. Third, other inflammatory conditions have not been discussed in the original study. Inflammatory control samples from patients without AEG are not included. Fourth, whether the stage of the tumor will affect the outcome is unknown. We also tried to further determine a possible effect through the subgroup analysis. However, on the basis of the available data, the subgroup analysis cannot be completed. Relative to more emergence, we can conduct additional analysis to further the value of NLR and PLR for AEG in different stages. Finally, meta-analysis is an observational study that may be limited by raw data bias and defects.

NLR is an inexpensive, easy-to-access, and multi-examination index. It is also an expected index for patients with AEG. This indicator helps identify high-risk patients and determine treatment plans. However, given the above limitations, NLR should be carefully used as a marker before it is recommended for clinical applications. The value of NLR in the prognosis of AEG should be verified before it is applied to clinical decision-making, and the value of PLR and LMR in AEG is worth further exploration.

## Data Availability Statement

The raw data supporting the conclusions of this article will be made available by the authors, without undue reservation, to any qualified researcher.

## Author Contributions

XL, QT, and SL conceived and designed this study. ZG and QZ searched and collected the data. XL and QZ contributed to data extraction and data analysis. QZ and BG performed the statistical analysis and interpretation of data. XL and FY wrote the manuscript. SP, ZG, and QT reviewed and revised the paper. XL and SL approved and submitted the final manuscript. All authors read, and approved the final manuscript and its submission.

### Conflict of Interest

The authors declare that the research was conducted in the absence of any commercial or financial relationships that could be construed as a potential conflict of interest.
